# Management of Melanoma Families 

**DOI:** 10.3390/cancers2020549

**Published:** 2010-04-16

**Authors:** Wilma Bergman, Nelleke A. Gruis

**Affiliations:** Department of Dermatology, Leiden University Medical Center, Leiden, The Netherlands

**Keywords:** : familial melanoma, hereditary melanoma, FAMMM syndrome, pancreatic carcinoma, management, surveillance, screening, DNA testing, CDKN2A

## Abstract

In this review we have aimed to focus on the clinical management of familial melanoma patients and their relatives. Along this line three major topics will be discussed: (1) management/screening of familial melanoma families: what is advised and what is the evidence thereof; (2) variability of families worldwide with regard to clinical phenotype, including cancer spectrum and likelihood of finding germline mutations and (3) background information for clinicians on the molecular biology of familial melanoma and recent developments in this field.

## 1. Introduction to the Clinical Phenotype and Diagnostic Criteria

Melanoma is hereditary in approximately 10% of the cases [1,2]. The diagnostic criteria are defined as the occurrence of invasive cutaneous melanoma in two or more first-degree family members, or three or more family members (irrespective of the degree of relationship) on the same side of the family. Familial melanoma is also known as Familial Atypical Multiple Mole-Melanoma (FAMMM) syndrome [3]. Although many members of melanoma families exhibit atypical nevi, the occurrence of clinical atypical nevi (CAN) is not required for the diagnosis [4]. However, the presence of multiple atypical nevi in family members means that they are three times more likely to be carrier of a mutation in a melanoma predisposing gene than their relatives without atypical moles [5].

**Figure 1:**
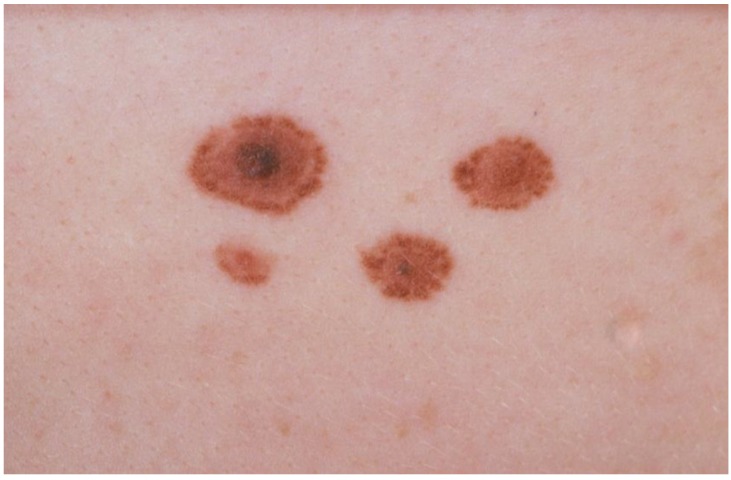
Classical example of an atypical nevus with asymmetry, color irregularity and a diameter of more than 5 mm.

Atypical nevi (formerly known as dysplastic nevi, named after their histological characteristics) can be defined as large, acquired melanocytic nevi, predominantly flat or macular, with several of the following features: asymmetry, indistinct borders, variation in pigmentation and a diameter of 5 mm or larger (Figure 1). There is no consensus on the definition of CAN. In some instances histological characteristics of dysplasia can be found in nevi smaller than 5 mm and in some instances no histological features of dysplasia can be found in moles fulfilling all clinical criteria of CAN mentioned above. Also, no international agreement exists on the histological criteria of dysplasia, although in most publications the same features are mentioned: loss of normal nevus architecture, increase of stromal tissue and inflammatory infiltrate and most important (some degree of) hyperplasia and cytological atypia of melanocytes [6,7].

It is important to realize that for international studies very strict definitions should be maintained for the sake of comparability. However, for patient care the definitions should be applied less strict and the concept of CAN should be envisioned as a continuum of increasing degrees of atypia that may stabilize at any point, or continue towards melanoma in rare cases [8,9].

Notwithstanding the above mentioned uncertainties it is after all very well possible to recognize hereditary melanoma families in clinical practice. Subsequently a screening program can be offered to those at increased risk and thereby early detection of melanoma can be realised [10,11]. Practical guidelines are being provided in the next part.

## 2. Management of FAMMM Families: Practical Guidelines and Review of the Evidence Thereof

The basis of recognizing familial clustering of melanoma is a family history to be obtained from all melanoma patients and all patients with multiple atypical moles. A rough drawing of a pedigree, during the family history, will help the clinician to oversee the structure of the pedigree and decide whether that family fulfills the diagnostic criteria. A checklist can be used to identify possible new melanoma-prone families (Table 1).

**Table 1:** Checklist to identify possible new melanoma-prone families.

Other family members with melanoma?	(1) degree: age Dx: (2) degree: age Dx: (3) degree: age Dx:
Youngest age at diagnosis of melanoma in the family	……….. years
Multiple primary melanomas in the family	Yes / No
Other cancer types in the family?	Pancreatic carcinoma Yes / No Other: …………………..
Atypical nevi in the patient?	Yes / No
Atypical nevi in first-degree relatives?	Yes / No
Skin type I and red hair in the family?	Yes / No
Reported cancer cases verified by medical documentation?	Yes / No
Referral to Clinical Geneticist	Yes / No
Likelihood of finding mutations in this family	Low / High

Age Dx: age at diagnosis

The ages at diagnosis of the melanomas as well as multiple melanoma (MM) cases should be indicated in the pedigree, as MM and melanomas before the age of 40, in the presence of a family history, are indicative of familiality for the disease. In addition the occurrence of other types of cancer, especially pancreatic carcinoma should be depicted in the pedigree. In countries where founder mutations are known to be associated with pancreatic carcinoma (see section on variability of FAMMM) the occurrence of this tumor might be included in the diagnostic criteria.

The next step is to extend the pedigree with all first- and second degree family members of melanoma patients to identify additional family members at increased risk. These family members should be advised to undergo a total body skin examination by a dermatologist and join regular screening programs. Despite the fact that dermatologists should be aware of hereditary melanoma, in many countries clinical geneticists are the appropriate specialists to validate the diagnosis. Apart from constructing concise medical pedigrees, access to pathology reports on tumors and identification of additional family members at increased risk belong to their specialty. In about a quarter of the families presented to the Dutch Familial Cancer Registry, cancer cases appear to be miscategorized by the patient after verification, jeopardizing the diagnosis of familial melanoma. Dermatologists, surgeons and other specialists dealing with melanoma patients might therefore better refer the patient to a department of Clinical Genetics. After the diagnosis of familial melanoma has been ascertained, in several countries the patient/family can seek genetic counseling and genetic testing. Because DNA testing melanoma cases are only informative that may be a problem in families in which all melanoma patients have died. Only in families with well known causal mutations predictive DNA testing is an option for family members who want to know their gene carrier status [12]. 

There is no evidence that all family members, regardless of the occurrence of atypical nevi should be included in regular screening programs. In the Leiden Pigmented Lesions Clinic (PLC) we have decided to include all first- and second degree family members in clinical management after we noticed that about 20% of our familial melanoma patients did neither show any atypical nevus nor any banal nevi at all. Inclusion of second degree relatives in regular screening protocols will significantly increase the workload of the PLC and therefore clinical studies about the efficacy of this practice are needed. 

There is no worldwide accepted policy for regular skin screenings and in some countries even legal considerations (lawsuits) might have influence on the frequency of examinations. At the Leiden PLC 90% of the patients are examined only once a year, the other 10% are advised to visit every 4–6 months because of very high numbers of atypical nevi. All patients (preferably in the presence of the partner) are instructed for skin self-examination (SSE) and advised to come back at once when they notice any change in an existing mole or a new mole after the age of 30. A total body mapping digital photo session [13] of all patients with atypical moles is performed and patients are provided with a CD with their photographs. The photographs are also stored on the hospital IT-system of the LUMC and can be used as a reference during each visit at the PLC. Due to the very variable phenotypes of the patients and the inclusion of first- and second degree relatives in the screening protocol, these photographs are actually reviewed in about 25% of the patients attending each clinic. The other patients show no worrisome moles that require serial digital examination.

Additional investigation of atypical moles with dermoscopy or other microscopic techniques is indispensable [14]. In patients at very high risk, in addition to clinically suspicious lesions, all changing moles, all symptomatic (itching, stinging, ‘active’) moles and all indistinct lesions are excised for histopathology. Excisions are always complete (*in toto*) to facilitate the histological investigation. 

As sun sensitivity (light skin type, red/blonde hair color) and exposure to UV radiation are additional risk factors for melanoma, members of melanoma families should be informed about these risks and advised to avoid sunburn and tanning activities. Epidemiologic observations suggest that in particular sunburns in childhood are associated with susceptibility to melanoma [15]. These points should be stressed in patient education, including educational leaflets.

## 3. Efficacy of Screening/Surveillance of FAMMM Families

Because little clinical research seems to have been published about the efficacy of screening/surveillance of FAMMM families the authors have sought to verify this impression and conducted a systematic review of the literature from the year 2000 till December 2009 (Table 2).

**Table 2:** Systematic review of the literature on management of familial melanoma.

	# hits	Relevant after screening title	Minus duplicate titles	Relevant after screening abstract
Search 1	153	54	37	25
Search 2	46	32	17	14
Search 3	146	62	30	17
Total	345	148	84	56

Systematic review of the literature (PubMed) published in English, French, German and Dutch on management of familial melanoma from January 2000 until December 2009, including the following search terminology: Search 1 = familial melanoma or hereditary melanoma or FAMMM syndrome or familial multiple atypical mole – melanoma syndrome Search 2 = search 1 × management or screening or surveillance Search 3 = melanoma/genetics × management or screening or surveillance minus hits in search 2.

Finally 56 publications (Table 3) remained of (some) interest, of which 25 were reviews or guidelines in which no new results were presented. These reviews did not actually ‘review’ research data on screening or management of familial melanoma families but rather mainly gave the opinion of experts in the field [12,16,17,18,19,20,21,22,23,24,25,26,27,28,29,30,31,32,33,34,35,36,37,38,39]. However, in evidence based medicine the opinion of experts is regarded as the lowest class of evidence. Of the 31 remaining publications nine reported on specific founder populations [40,41,42,43,44,45,46,47,48]. None of these publications reported follow up or management data. 

**Table 3:** Fifty-six key-references with regard to management of familial melanoma published after 1 January 2000.

Titles on management of familial melanoma	N = 56	References:
Reviews/guidelines	N = 25	[12,16,17,18,19,20,21,22,23,24,25,26,27,28,29,30,31,32,33,34,35,36,37,38,39]
National reports/founder populations	N = 9	[40,41,42,43,44,45,46,47,48]
Regarding (preventive) DNA testing	N = 8	[49,50,51,52,53,54,55,56]
Regarding pancreatic carcinoma (risk/screening)	N = 5	[57,58,59,60,61]
Clinical features: sun sensitivity, early onset, age, metastasis, body site, survival, MC1R variants	N = 5	[62,63,64,65,66]
Management/screening	N = 4	[4,67,68,69]

Eight papers dealt with (predictive) DNA diagnosis. Leachman *et al.* have tried to identify the small proportion of melanoma patients who benefit most from DNA diagnosis. Because of the variability in the rate of CDKN2A mutations they advocate different approaches in different countries. Except in regions of high melanoma incidence (Australia) they advise testing in individuals with three or more primary melanomas and in melanoma patients from families with at least one invasive melanoma and two or more other invasive melanomas or pancreatic cancers on the same side of the family [49]. Kasparian *et al.* explored the uptake of predictive DNA testing in an Australian population of melanoma patients with varying degrees of hereditary melanoma risk [50,51]. They concluded that individuals at highest risk, females more than males, were most interested in predictive DNA testing. They stressed the importance of identifying misconceptions that may impede decision making about genetic testing. De Snoo *et al.* and Riedijk *et al.* also showed that the motivation of those likely to decline testing appeared to be associated with disease misperceptions [52,53]. 

In general DNA testing in hereditary melanoma does not seem to be associated with worrisome levels of distress, as measured after test reporting and counselling [54,55]. After a positive DNA diagnosis the compliance of early melanoma detection behaviors increased in a study from Utah [56].

Five papers reported on the risk of pancreatic carcinoma in FAMMM families and discussed that families with mutations in the major melanoma gene CDKN2A should be considered for pancreatic cancer surveillance [57,58,59,60,61]. Also, with respect to pancreatic carcinoma no evidence for screening guidelines does exist. The best method to find pancreatic carcinoma in an early phase is unknown. Two studies from the Netherlands report that screening with endoscopic ultrasonography is feasible and safe. They found early asymptomatic lesions in the pancreas in 7% of their study population [61]. However, whether screening improves survival remains to be determined. Research on ‘patient friendly’ ways for early diagnosis of pancreatic carcinoma (e.g., detection of tumour associated markers in serum or stool) is badly needed for surveillance of CDKN2A positive families.

Five papers dealt with phenotypic or environmental factors relevant to management of melanoma families. Briollais *et al.* reported that melanoma families irrespective of their gene status show a significant aggregation of the following melanoma risk factors: large number of nevi, light phototype and a high degree of sun exposure [62]. They concluded that familial melanoma may be the result of an accumulation of several genetic factors in the absence of a known major melanoma-associated gene defect. This clinical observation has been supported by a study in Sweden where MC1R variants (red hair, light skin) were found to be associated with an increased risk of melanoma in members of hereditary melanoma families [63]. 

Hornbuckle *et al.* investigated patterns of metastasis in familial and non-familial melanoma and found no significant differences in two groups of 38 familial cases and 114 matched non-familial melanoma cases with metastatic disease [64]. This means that familial melanoma patients do not need to undergo a more vigorous oncological follow-up program for their melanomas.

In familial and non-familial melanoma patients with multiple primaries, Gillgren *et al.* investigated differences in the body distribution of melanomas [65]. This study revealed that familial melanomas were found significantly less on the head and neck than on the trunk. Florell *et al.* Combined the Mormon pedigrees with the Utah cancer registry [66] and concluded that prognostic factors like gender, tumour thickness and level of invasion do not differ between familial and non-familial melanomas, and that there is no difference in survival. They also concluded that familial melanoma does not seem to have a significantly different biologic behaviour. 

After all, four papers remained in which results relevant to screening of melanoma families were described [4,67,68,69]. An American study showed that the mean tumour thickness of prospectively identified melanomas (*i.e.*, by means of screening activities) was 0.46 mm and of those identified prior to the study 1.03 mm (in 17 CDKN2A positive families) thus indirectly illustrating the efficacy of screening [4].

A Swedish group has evaluated a program in which 280 melanoma families were followed for 14 years between 1987 and 2001 [67]. Only 69 percent of these families fulfilled the criteria for hereditary melanoma (as mentioned in the introduction above). In this study the median tumour thickness of the 41 ‘hereditary’ melanomas was significantly thinner compared to the median tumour thickness of the general population. The median tumour thickness of 26 invasive melanomas was 0.5 mm and 15 (37%) were melanomas *in situ*. Sixty-six percent of all melanomas discovered during the study were diagnosed in patients who had had at least one previous melanoma. In addition, melanoma patients had a significantly more sun sensitive skin type and a higher number of atypical moles as compared to non-affected family members [67]. 

Instruction for skin self examination (SSE) is an important part of screening high risk patients. Motivational factors for the performance of SSE in a cohort of familial melanoma patients were investigated [68]. One third of this high risk population did not report an adequate frequency of SSE. Adequate performers of SSE were more likely to have a partner, had a more positive attitude towards SSE and perceived SSE less difficult to perform compared to poor performers. Clearly families with a history of melanoma may benefit from knowing about their risk and behaviours to modify that risk. The purpose of one study was to qualitatively describe intra familial risk communication in relation to screening to generate factors that might hamper early diagnosis of melanoma [69]. We can learn from this study that most families had a high awareness of their melanoma susceptibility, discuss risk behaviours with each other and feel obliged to tell others to minimize sun exposure [69]. 

In conclusion, although it is generally advised to invite members of melanoma prone families to regular skin examinations, no evidence exists on several aspects of such a screening program. It is thought that members of melanoma prone families should be followed up with total body photography, with or without dermoscopy pictures of atypical nevi; however this is an elaborate approach, which requires the availability of a photographer and a nurse. Still the efficacy of it remains to be proven. Furthermore there is neither evidence of the efficacy of including second degree family members or family members without (atypical) moles, nor about the age to start (melanomas before puberty are extremely rare) or end (at age 60, 70 never having had melanoma). Psychological, motivational and communicational aspects of familial melanoma are sparse as well.

## 4. FAMMM: Phenotype and Genetic Variability

The FAMMM syndrome exhibits variability in almost all aspects of the phenotype: penetrance of the melanoma trait, the presence of atypical nevi, association with other tumor types, the age of youngest diagnosis of melanoma, the percentage and number of multiple primary melanomas, the likelihood of finding mutations, *etc*.

The chance of finding mutations in CDKN2A in melanoma families was examined in 385 families worldwide and found to vary widely across continents [70]. The frequency of mutations increased significantly as the number of melanoma cases in the family increased, with the highest likelihood of finding mutations in ≥6 case families in Australia, ≥5 case families in the USA and ≥4 case families in Europe. The frequency of mutations also depended on the number of multiple melanoma cases in the family and a low median age of melanoma diagnosis. Also these variables differed in a predictable direction between the continents: in Australia more multiple primary melanoma cases and lower median age at diagnosis were needed to increase the likelihood of finding a mutation [70]. In Italy the likelihood to identify CDKN2A mutations in families with two melanoma cases was 25% [71].

Bishop *et al.* modeled penetrance for melanoma in 80 families with mutations in the major melanoma gene CDKN2A, and an average of five melanoma patients per family [72]. Overall CDKN2A mutation penetrance was 0.30 (95% CI 0.12–0.62) by age 50 and 0.67 (95% CI 0.31–0.96) by age 80. Penetrance estimates differed according to the population incidence rate of melanoma: by age 80 years 0.58 in Europe, 0.76 in the USA and 0.91 in Australia [72]. The GEM study group studied lifetime risk of melanoma in CDKN2A mutation carriers present in a population-based sample and estimated the penetrance of melanoma in CDKN2A carriers as 0.28 (95% CI 0.18–0.40). It thus appeared that CDKN2A mutation carriers in the general population have a lower risk of melanoma compared to carriers from multiple case families, which is probably due to the co-existence of genetic variants that affect risk [73].

Although pancreatic carcinoma was described in earlier reports of familial melanoma kindred in the USA [3], the first association that CDKN2A mutations also predispose patients to pancreatic carcinoma was established in Dutch melanoma families harboring a founder mutation in CDKN2A, known as P16-Leiden deletion [74,75,76,77]. Nowadays this association is recognized in many families with CDKN2A mutations worldwide [57,77], not only for mutations affecting the p16 transcript of the CDKN2A gene but also the p14ARF transcript, albeit to a lesser extend [78]. The melanoma genetics consortium (Genome) studied the relationship between pancreatic cancer and familial melanoma and found that the occurrence of pancreatic carcinoma significantly predicted the likelihood of finding CDKN2A mutations, with the exception of Australia [70]. In Australia the spectrum of mutations seems to be different from Europe and the USA. It has not been possible to attribute pancreatic cancer to certain CDKN2A mutations yet. 

A risk analysis in 19 Dutch P16-Leiden families with 656 relatives revealed a cumulative risk of developing pancreatic cancer in assumed mutation carriers by age 75 of 17% [79]. Pancreatic carcinomas were found in seven of these 19 families. The mean age at diagnosis was 58 years (range 38–77), which is seven years earlier than the age of diagnosis of unselected cases of pancreatic carcinoma. Lynch *et al.* approached the subject from the perspective of familial pancreatic carcinoma and reported 12% of their pancreatic carcinoma families to be associated with the FAMMM cutaneous phenotype in association with a CDKN2A mutation [59]. These families showed, besides early onset pancreatic carcinoma several other types of cancer such as cancers of the upper digestive tract (in particular esophageal cancer), breast and lung cancer. These associations have been attributed to an ascertainment bias (family selection at a hereditary cancer clinic) by many investigators; however the broad tumor spectrum could be confirmed in Dutch P16-Leiden families which had been ascertained at a pigmented lesions clinic [79]. This analysis included 1,528 individuals and over 35,000 person years of follow-up and showed a significant increased risk of non-melanoma skin cancer (RR 24), tumors of the oral cavity (RR 7.8), lung cancer (RR 5.6) and breast cancer (RR 2.1) in mutation carriers. Brain tumors were also found in excess [79]. 

## 5. High Risk Melanoma Gene Search Reflects Complexity

The search for familial melanoma genes started already decades ago. Linkage initiatives in small numbers of families undertaken by several genetic research laboratories painfully showed that identification of high risk melanoma genes is difficult, probably reflecting the complex polygenetic nature of the disease. Only one initiative in well described families from Utah and Texas revealed a locus from which a melanoma predisposing gene was cloned [80,81]. This CDKN2A gene turned out to be an important cell cycle regulator with a key tumour suppressor role acting in the gestation or promotion of several cancer types [81].

CDKN2A encodes for two disparate proteins p16 (exons1α, 2, and 3) and p14ARF (exons 1β, 2 and 3) [82]. P16 is a cyclin-dependent kinase inhibitor with cell-cycle control functions and an inducer of senescence of melanocytes [83,84]. P14ARF blocks HDM2 inhibition of p53 activity, so that this locus impacts on two key pathways in cancer, the retinoblastoma pathway and the p53 pathway [82]. In addition, two other functions have been attributed to p14ARF: sumoylation of several of its binding partners [85] and regulation of ribosome biogenesis [86]. The majority of the germline mutations reported for familial melanoma are present in CDKN2A exon 2 and impact most of the time on both proteins. Mutations in exon 1α impact on p16 alone and those in exon 1β on p14ARF alone. Since mutations occurring in exon 1α or exon 2 display no apparent difference in the cancer pattern in families and since the percentage of mutations affecting exon 1β is still low (2%) [87], it is surmised that p16 protein is key. However, currently there is a slight trend that hereditary mutations at the locus impacting on p14ARF are related to the occurrence of neural system tumours in melanoma families [88].

A second melanoma susceptibility gene, CDK4, was identified by candidate gene approach. Very small numbers of families (2%) have hereditary mutations in this gene, which encodes for the p16 binding site. The mutations restrict themselves so far to two missense mutations at codon 24 in exon 2 of the CDK4 gene resulting in amino acid changes Arg24Cys and Arg24His [89,90].

Worldwide the chance of finding a mutation in these two recognised high risk melanoma genes in families with three or more cases of melanoma is approximately 40% [70]. The possibility that the missing mutations or inactivation of CDKN2A in remaining families can be explained by deletion or epi-mutation of the CDN2A locus respectively, has been excluded [91,92]. Absence of mutations at the CDKN2A or CDK4 loci, even in families with very large numbers of melanoma cases therefore indicate other yet to be identified high penetrance susceptibility genes. Evidence for a new locus on chromosome 1p22 was already published seven years ago, but a new melanoma gene has not yet been identified [93].

In the search for additional genetic risk factors for melanoma it appeared that polymorphisms in genes controlling skin colour act as risk factors for melanoma [94,95]; a finding that is not that surprising since melanoma is especially a disease in white skinned individuals. 

Skin colour is a result of melanin synthesis commenced with the binding of the melanocyte-stimulating hormone (αMSH) to the melanocortin-1 receptor (MC1R) [96]. The subsequent signalling cascade acting through the secondary messenger adenylate cyclase results in up regulation of tyrosinase. The initiation of these events produces a series of spontaneous and catalytic reactions ultimately resulting in the production of brown/black eumelanin. Another ligand of MC1R is the agouti signalling protein (ASIP). Binding of ASIP to MC1R results in the blockage of the αMSH signalling cascade, then ineffective eumelanin production [97]. Produced pigment is transported within melanosomes. P protein (OCA2) plays an important role in melanosome biogenesis, and controls the eumelanin content in melanocytes [98], in part via the processing and trafficking of tyrosinase—the rate limiting enzyme in melanin synthesis [99]. The fact that variants in the MC1R receptor gene control, at least in part, some of melanoma risk factors such as red hair [100,101] and freckles [102], identify MC1R as a candidate probably low risk melanoma susceptibility gene. Its role as such has become even more apparent after performing recent genome wide single nucleotide polymorphism (SNP) association studies in a huge melanoma case-control cohort [103]. 

Genome wide association studies allow much more powerful approaches to the identification of low to medium penetrance susceptibility genes. Of interest is that SNPs in or near genes previously identified as associated with sun-sensitive phenotypes, such as variants near the ASIP locus (which codes for the agouti protein), the tyrosinase locus (TYR) and tyrosinase related protein 1 (TYRP1) are now identified as melanoma susceptibility genes [103,104,105]. Of particular interest however is that a “hit” on chromosome 9, near to *CDKN2A*, is associated both with melanoma risk and nevus number [106]. A second nevus locus was identified on chromosome 22 and this was the first clear evidence of nevus genes from genome wide studies. These exciting developments have the potential to identify the biological implications of these genes and to better understand how melanoma develops.

## 6. Conclusions

Melanoma families have been identified worldwide, but joint efforts have only revealed major melanoma-associated gene mutations in less than halve of the families, mostly CDKN2A mutations, involving two cell cycle controlling proteins called p16 and p14ARF. The chance of finding mutations was found to vary widely across continents, with chances of finding mutations in 2 case families and in multiple melanoma patients being low. 

Additional genetic risk factors for melanoma appeared to be polymorphisms in genes controlling skin color, positioned in the melanocortin-1 receptor pathway. It is thought that combinations of several of these low-risk melanoma associated gene mutations may be responsible for familial clustering of melanomas as well.

Pancreatic carcinoma appears to be part of the tumor spectrum associated with several CDKN2A mutations worldwide. Risk estimates revealed a cumulative risk of 17% by age 75. 

Guidelines for screening members of melanoma prone families are primarily based on expert opinions, not on significant evidence from clinical research. It is generally advised to invite members of melanoma prone families to join regular skin examinations and to use total body photography and serial dermoscopy to facilitate screening. Patients should be instructed for skin self examination. All countries should aim to develop national guidelines for screening and set indications for genetic counseling and DNA testing of members of melanoma families. 

## References

[1] Ford D., Bliss J.M., Swerdlow A.J., Armstrong B.K., Franceschi S., Green A., Holly E.A., Mack T., MacKie R.M., Osterlind A. (1995). Risk of cutaneous melanoma associated with a family history of the disease. The International Melanoma Analysis Group (IMAGE). Int. J. Cancer.

[2] Goldstein A.M., Tucker M.A. (1995). Genetic epidemiology of familial melanoma. Dermatol. Clin..

[3] Lynch H.T., Frichot B.C., Lynch J.F. (1978). Familial atypical multiple mole-melanoma syndrome. J. Med. Genet..

[4] Tucker M.A., Fraser M.C., Goldstein A.M., Struewing J.P., King M.A., Crawford J.T., Chiazze E.A., Zametkin D.P., Fontaine L.S., Clark W.H. (2002). A natural history of melanomas and dysplastic nevi: an atlas of lesions in melanoma-prone families. Cancer.

[5] Bishop J.A., Wachsmuth R.C., Harland M., Bataille V., Pinney E., Mack P., Baglietto L., Cuzick J., Bishop D.T. (2000). Genotype/phenotype and penetrance studies in melanoma families with germline CDKN2A mutations. J. Invest. Dermatol..

[6] Clark W.H., Tucker M.A. (1998). Problems with lesions related to the development of malignant melanoma: common nevi, dysplastic nevi, malignant melanoma *in situ*, and radial growth phase malignant melanoma. Hum. Pathol..

[7] Rabkin M.S. (2008). The limited specificity of histological examination in the diagnosis of dysplastic nevi. J. Cutan. Pathol..

[8] Hussein M.R. (2005). Melanocytic dysplastic naevi occupy the middle ground between benign melanocytic naevi and cutaneous malignant melanomas: emerging clues. J. Clin. Pathol..

[9] Miller A.J., Mihm M.C. (2006). Melanoma. N. Engl. J. Med..

[10] Vasen H.F., Bergman W., van Haeringen A., Scheffer E., van Slooten E.A. (1989). The familial dysplastic nevus syndrome. Natural history and the impact of screening on prognosis. A study of nine families in the Netherlands. Eur. J. Cancer Clin. Oncol..

[11] Masri G.D., Clark W.H., Guerry D., Halpern A., Thompson C.J., Elder D.E. (1990). Screening and surveillance of patients at high risk for malignant melanoma result in detection of earlier disease. J. Am. Acad. Dermatol..

[12] de Snoo F.A., Bergman W., Gruis N.A. (2003). Familial melanoma: a complex disorder leading to controversy on DNA testing. Fam. Cancer.

[13] Halpern A.C., Marghoob A.A., Bialoglow T.W., Witmer W., Slue W. (2003). Standardized positioning of patients (poses) for whole body cutaneous photography. J. Am. Acad. Dermatol..

[14] Bafounta M.L., Beauchet A., Aegerter P., Saiag P. (2001). Is dermoscopy (epiluminescence microscopy) useful for the diagnosis of melanoma? Results of a meta-analysis using techniques adapted to the evaluation of diagnostic tests. Arch. Dermatol..

[15] Whiteman D.C., Whiteman C.A., Green A.C. (2001). Childhood sun exposure as a risk factor for elanoma: a systematic review of epidemiologic studies. Cancer Causes Contr..

[16] Hershock D. (2005). Genetics, prevention and screening for melanoma. Cancer Chemother. Biol. Response Modif..

[17] Hayward N. (2000). New developments in melanoma genetics. Curr. Oncol. Rep..

[18] Kefford R.F., Mann G.J. (2003). Is there a role for genetic testing in patients with melanoma?. Curr. Opin. Oncol..

[19] Rivers J.K. (2004). Is there more than one road to melanoma?. Lancet.

[20] Lynch H.T., Fusaro R.M., Lynch J.F. (2007). Hereditary cancer syndrome diagnosis: molecular genetic clues and cancer control. Future Oncol..

[21] Bishop J.N., Harland M., Randerson-Moor J., Bishop D.T. (2007). Management of familial melanoma. Lancet Oncol..

[22] Dalle S., Martin-Denavit T., Thomas L. (2006). Genotypic hypervariability of melanoma: a therapeutic challenge. Med. Sci..

[23] Lange J.R., Balch C.M. (2005). Screening for cutaneous melanoma. Surg. Oncol. Clin. N. Am..

[24] Tsao H., Niendorf K. (2004). Genetic testing in hereditary melanoma. J. Am. Acad. Dermatol..

[25] Hansen C.B., Wadge L.M., Lowstuter K., Boucher K., Leachman S.A. (2004). Clinical germline genetic testing for melanoma. Lancet Oncol..

[26] Czajkowski R., Placek W., Drewa G., Czajkowska A., Uchanska G. (2004). FAMMM syndrome: pathogenesis and management. Dermatol. Surg..

[27] Gibbs P., Brady B.M., Robinson W.A. (2002). The genes and genetics of malignant melanoma. J. Cutan. Med. Surg..

[28] Gruis N.A., Bergman W. (2000). From gene to disease; from p16 to melanoma. Ned. Tijdschr. Geneeskd..

[29] Fusaro R.M., Lynch H.T. (2000). The FAMMM syndrome: epidemiology and surveillance strategies. Cancer Invest..

[30] Itin P.H. (2000). Skin check-up—who and when?. Ther. Umsch..

[31] Platz A., Ringborg U., Hansson J. (2000). Hereditary cutaneous melanoma. Semin. Cancer Biol..

[32] Oliveria S., Dusza S., Berwick M. (2001). Issues in the epidemiology of melanoma. Expert. Rev. Anticancer Ther..

[33] Tucker M.A., Goldstein A.M. (2003). Melanoma etiology: where are we?. Oncogene.

[34] Stahl J.M., Sharma A., Cheung M., Zimmerman M., Cheng J.Q., Bosenberg M.W., Kester M., Sandirasegarane L., Robertson G.P. (2004). Deregulated Akt3 activity promotes development of malignant melanoma. Cancer Res..

[35] Pho L., Grossman D., Leachman S.A. (2006). Melanoma genetics: a review of genetic factors and clinical phenotypes in familial melanoma. Curr. Opin. Oncol..

[36] Kefford R., Bishop J.N., Tucker M., Bressac-de P.B., Bianchi-Scarra G., Bergman W., Goldstein A., Puig S., Mackie R., Elder D., Hansson J., Hayward N., Hogg D., Olsson H. (2002). Genetic testing for melanoma. Lancet Oncol..

[37] Fraser M.C., Goldstein A.M., Tucker M.A. (2004). Genetic testing for inherited predisposition to melanoma: has the time come?. J. Drugs Dermatol..

[38] Hansson J. (2008). Familial melanoma. Surg. Clin. North Am..

[39] Santillan A.A., Cherpelis B.S., Glass L.F., Sondak V.K. (2009). Management of familial melanoma and nonmelanoma skin cancer syndromes. Surg. Oncol. Clin. North. Am..

[40] Mantelli M., Pastorino L., Ghiorzo P., Barile M., Bruno W., Gargiulo S., Sormani M.P., Gliori S., Vecchio S., Ciotti P., Sertoli M.R., Queirolo P., Goldstein A.M., Bianchi-Scarra G. (2004). Early onset may predict G101W CDKN2A founder mutation carrier status in Ligurian melanoma patients. Melanoma Res..

[41] Eldon B.J., Thorlacius S., Jonsson T., Jonasson J.G., Kjartansson J., Bodvarsson S., Steingrimsson E., Rafnar T. (2006). A population-based study on the familial aggregation of cutaneous malignant melanoma in Iceland. Eur. J. Cancer.

[42] Lamperska K., Karezewska A., Kwiatkowska E., Mackiewicz A. (2002). Analysis of mutations in the p16/CDKN2A gene in sporadic and familial melanoma in the Polish population. Acta Biochim. Pol..

[43] Pjanova D., Engele L., Randerson-Moor J.A., Harland M., Bishop D.T., Newton Bishop J.A., Taylor C., Debniak T., Lubinski J., Kleina R., Heisele O. (2007). CDKN2A and CDK4 variants in Latvian melanoma patients: analysis of a clinic-based population. Melanoma Res..

[44] Ashton-Prolla P., Bakos L., Junqueira G., Giugliani R., Azevedo S.J., Hogg D. (2008). Clinical and molecular characterization of patients at risk for hereditary melanoma in southern Brazil. J. Invest. Dermatol..

[45] Gensini F., Sestini R., Piazzini M., Vignoli M., Chiarugi A., Brandani P., Ghiorzo P., Salvini C., Borgognoni L., Palli D., Bianchi-Scarra G., Carli P., Genuardi M. (2007). The p.G23S CDKN2A founder mutation in high-risk melanoma families from Central Italy. Melanoma Res..

[46] Borges A.L., Cuellar F., Puig-Butille J.A., Scarone M., Delgado L., Badenas C., Mila M., Malvehy J., Barquet V., Nunez J., Laporte M., Fernandez G., Levrero P., Martinez-Asuaga M., Puig S. (2009). CDKN2A mutations in melanoma families from Uruguay. Br. J. Dermatol..

[47] Peric B., Cerkovnik P., Novakovic S., Zgajnar J., Besic N., Hocevar M. (2008). Prevalence of variations in melanoma susceptibility genes among Slovenian melanoma families. Med. Genet..

[48] Nagore E., Botella-Estrada R., Garcia-Casado Z., Requena C., Serra-Guillen C., Llombart B., Sanmartin O., Guillen C. (2008). Comparison between familial and sporadic cutaneous melanoma in Valencia, Spain. J. Eur. Acad. Dermatol. Venereol..

[49] Leachman S.A., Carucci J., Kohlmann W., Banks K.C., Asgari M.M., Bergman W., Bianchi-Scarra G., Brentnall T., Bressac-de P.B., Bruno W., Curiel-Lewandrowski C., de Snoo F.A., Debniak T., Demierre M.F., Elder D., Goldstein A.M., Grant-Kels J., Halpern A.C., Ingvar C., Kefford R.F., Lang J., MacKie R.M., Mann G.J., Mueller K., Newton-Bishop J., Olsson H., Petersen G.M., Puig S., Rigel D., Swetter S.M., Tucker M.A., Yakobson E., Zitelli J.A., Tsao H. (2009). Selection criteria for genetic assessment of patients with familial melanoma. J. Am. Acad. Dermatol..

[50] Kasparian N.A., Meiser B., Butow P.N., Soames Job R.F., Mann G.J. (2007). Anticipated uptake of genetic testing for familial melanoma in an Australian sample: An exploratory study. Psychooncology..

[51] Kasparian N.A., Butow P.N., Meiser B., Mann G.J. (2008). High- and average-risk individuals' beliefs about, and perceptions of, malignant melanoma: an Australian perspective. Psychooncology..

[52] Riedijk S.R., de Snoo F.A., van Dijk S., Bergman W., van Haeringen A., Silberg S., van Elderen T.M., Tibben A. (2005). Hereditary melanoma and predictive genetic testing: why not? Psychooncology.

[53] de Snoo F.A., Riedijk S.R., van Mil A.M., Bergman W., ter Huurne J.A., Timman R., Bertina W., Gruis N.A., Vasen H.F., van Haeringen A., Breuning M.H., Tibben A. (2008). Genetic testing in familial melanoma: uptake and implications. Psychooncology.

[54] Kasparian N.A., Meiser B., Butow P.N., Simpson J.M., Mann G.J. (2008). Predictors of psychological distress among individuals with a strong family history of malignant melanoma. Clin. Genet..

[55] Bergenmar M., Hansson J., Brandberg Y. (2009). Family members' perceptions of genetic testing for malignant melanoma—a prospective interview study. Eur. J. Oncol. Nurs..

[56] Aspinwall L.G., Leaf S.L., Dola E.R., Kohlmann W., Leachman S.A. (2008). CDKN2A/p16 genetic test reporting improves early detection intentions and practices in high-risk melanoma families. Cancer Epidemiol. Biomarkers Prev..

[57] Parker J.F., Florell S.R., Alexander A., DiSario J.A., Shami P.J., Leachman S.A. (2003). Pancreatic carcinoma surveillance in patients with familial melanoma. Arch. Dermatol..

[58] Bishop J.A., Wachsmuth R.C., Harland M., Bataille V., Pinney E., Mac K.P., Baglietto L., Cuzick J., Bishop D.T. (2000). Genotype/Phenotype and penetrance studies in melanoma families with germline CDKN2A mutations. J. Invest. Dermatol.

[59] Lynch H.T., Brand R.E., Hogg D., Deters C.A., Fusaro R.M., Lynch J.F., Liu L., Knezetic J., Lassam N.J., Goggins M., Kern S. (2002). Phenotypic variation in eight extended CDKN2A germline mutation familial atypical multiple mole melanoma-pancreatic carcinoma-prone families: the familial atypical mole melanoma-pancreatic carcinoma syndrome. Cancer.

[60] Kluijt I., Cats A., Fockens P., Nio Y., Gouma D.J., Bruno M.J. (2009). Atypical Familial Presentation of FAMMM Syndrome With a High Incidence of Pancreatic Cancer: Case Finding of Asymptomatic Individuals by EUS Surveillance. J. Clin. Gastroenterol..

[61] Poley J.W., Kluijt I., Gouma D.J., Harinck F., Wagner A., Aalfs C., van Eijck C.H., Cats A., Kuipers E.J., Nio Y., Fockens P., Bruno M.J. (2009). The yield of first-time endoscopic ultrasonography in screening individuals at a high risk of developing pancreatic cancer. Am. J. Gastroenterol..

[62] Briollais L., Chompret A., Guilloud-Bataille M., Bressac-de P.B., Avril M.F., Demenais F. (2000). Patterns of familial aggregation of three melanoma risk factors: great number of naevi, light phototype and high degree of sun exposure. Int. J. Epidemiol..

[63] Hoiom V., Tuominen R., Kaller M., Linden D., Ahmadian A., Mansson-Brahme E., Egyhazi S., Sjoberg K., Lundeberg J., Hansson J. (2009). MC1R variation and melanoma risk in the Swedish population in relation to clinical and pathological parameters. Pigment Cell Melanoma Res..

[64] Hornbuckle J., Culjak G., Jarvis E., Gebski V., Coates A., Mann G., Kefford R. (2003). Patterns of metastases in familial and non-familial melanoma. Melanoma Res..

[65] Gillgren P., Brattstrom G., Frisell J., Palmgren J., Ringborg U., Hansson J. (2003). Body site of cutaneous malignant melanoma--a study on patients with hereditary and multiple sporadic tumours. Melanoma Res..

[66] Florell S.R., Boucher K.M., Garibotti G., Astle J., Kerber R., Mineau G., Wiggins C., Noyes R.D., Tsodikov A., Cannon-Albright L.A., Zone J.J., Samlowski W.E., Leachman S.A. (2005). Population-based analysis of prognostic factors and survival in familial melanoma. J. Clin. Oncol..

[67] Hansson J., Bergenmar M., Hofer P.A., Lundell G., Mansson-Brahme E., Ringborg U., Synnerstad I., Bratel A.T., Wennberg A.M., Rosdahl I. (2007). Monitoring of kindreds with hereditary predisposition for cutaneous melanoma and dysplastic nevus syndrome: results of a Swedish preventive program. J. Clin. Oncol..

[68] Mesters I., Jonkman L., Vasen H. (2009). Skin self-examination of persons from families with familial atypical multiple mole melanoma (FAMMM). Patient Educ. Couns..

[69] Loescher L.J., Crist J.D., Siaki L.A. (2009). Perceived intrafamily melanoma risk communication. Cancer Nurs..

[70] Goldstein A.M., Chan M., Harland M., Hayward N.K., Demenais F., Bishop D.T., Azizi E., Bergman W., Bianchi-Scarra G., Bruno W., Calista D., Albright L.A., Chaudru V., Chompret A., Cuellar F., Elder D.E., Ghiorzo P., Gillanders E.M., Gruis N.A., Hansson J., Hogg D., Holland E.A., Kanetsky P.A., Kefford R.F., Landi M.T., Lang J., Leachman S.A., MacKie R.M., Magnusson V., Mann G.J., Bishop J.N., Palmer J.M., Puig S., Puig-Butille J.A., Stark M., Tsao H., Tucker M.A., Whitaker L., Yakobson E. (2007). Features associated with germline CDKN2A mutations: a GenoMEL study of melanoma-prone families from three continents. J. Med. Genet..

[71] Bruno W., Ghiorzo P., Battistuzzi L., Ascierto P.A., Barile M., Gargiulo S., Gensini F., Gliori S., Guida M., Lombardo M., Manoukian S., Menin C., Nasti S., Origone P., Pasini B., Pastorino L., Peissel B., Pizzichetta M.A., Queirolo P., Rodolfo M., Romanini A., Scaini M. C., Testori A., Tibiletti M.G., Turchetti D., Leachman S.A., Bianchi-Scarra G. (2009). Clinical genetic testing for familial melanoma in Italy: a cooperative study. J. Am. Acad. Dermatol..

[72] Bishop D.T., Demenais F., Goldstein A.M., Bergman W., Bishop J.N., Bressac-de P.B., Chompret A., Ghiorzo P., Gruis N., Hansson J., Harland M., Hayward N., Holland E.A., Mann G.J., Mantelli M., Nancarrow D., Platz A., Tucker M.A. (2002). Geographical variation in the penetrance of CDKN2A mutations for melanoma. J. Natl. Cancer Inst..

[73] Begg C.B., Orlow I., Hummer A.J., Armstrong B.K., Kricker A., Marrett L.D., Millikan R.C., Gruber S.B., Anton-Culver H., Zanetti R., Gallagher R.P., Dwyer T., Rebbeck T.R., Mitra N., Busam K., From L., Berwick M. (2005). Lifetime risk of melanoma in CDKN2A mutation carriers in a population-based sample. J. Natl. Cancer Inst..

[74] Vasen H.F., Gruis N.A., Frants R.R., van der Velden P.A., Hille E.T., Bergman W. (2000). Risk of developing pancreatic cancer in families with familial atypical multiple mole melanoma associated with a specific 19 deletion of p16 (p16-Leiden). Int. J. Cancer.

[75] de Vos tot Nederveen Cappel W.H., Offerhaus G.J., van P.M., Caspers E., Gruis N.A., de Snoo F.A., Lamers C.B., Griffioen G., Bergman W., Vasen H.F., Morreau H. (2003). Pancreatic carcinoma in carriers of a specific 19 base pair deletion of CDKN2A/p16 (p16-leiden). Clin. Cancer Res..

[76] Hille E., van Duijn E., Gruis N.A., Rosendaal F.R., Bergman W., Vandenbroucke J.P. (1998). Excess cancer mortality in six Dutch pedigrees with the familial atypical multiple mole-melanoma syndrome from 1830 to 1994. J. Invest. Dermatol..

[77] Lynch H.T., Fusaro R.M., Albano W.A., Pester J., Kimberling W.J., Lynch J.F. (1983). Phenotypic variation in the familial atypical multiple mole-melanoma syndrome (FAMMM). J. Med. Genet..

[78] Goldstein A.M., Chan M., Harland M., Gillanders E.M., Hayward N.K., Avril M.F., Azizi E., Bianchi-Scarra G., Bishop D.T., Bressac-de P.B., Bruno W., Calista D., Cannon Albright L.A., Demenais F., Elder D.E., Ghiorzo P., Gruis N.A., Hansson J., Hogg D., Holland E.A., Kanetsky P.A., Kefford R.F., Landi M.T., Lang J., Leachman S.A., Mackie R.M., Magnusson V., Mann G.J., Niendorf K., Newton B.J., Palmer J.M., Puig S., Puig-Butille J.A., de Snoo F.A., Stark M., Tsao H., Tucker M.A., Whitaker L., Yakobson E. (2006). High-risk melanoma susceptibility genes and pancreatic cancer, neural system tumors, and uveal melanoma across GenoMEL. Cancer Res..

[79] de Snoo F.A., Bishop D.T., Bergman W., van Leeuwen-Cornelissen I, van der Drift C., van Nieuwpoort F.A., Out-Luiting C.J., Vasen H.F., ter Huurne J.A., Frants R.R., Willemze R., Breuning M.H., Gruis N.A. (2008). Increased risk of cancer other than melanoma in CDKN2A founder mutation (p16-Leiden)-positive melanoma families. Clin. Cancer Res..

[80] Cannon-Albright L.A., Goldgar D.E., Neuhausen S., Gruis N.A., Anderson D.E., Lewis C. M., Jost M., Tran T.D., Nyguen K., Kamb A. (1994). Localization of the 9p melanoma susceptibility locus (MLM) to a 2-cM region between D9S736 and D9S171. Genomics.

[81] Kamb A., Gruis N.A., Weaver-Feldhaus J., Liu Q., Harshman K., Tavtigian S.V., Stockert E., Day R.S., Johnson B.E., Skolnick M.H. (1994). A cell cycle regulator potentially involved in genesis of many tumor types. Science.

[82] Chin L., Pomerantz J., DePinho R.A. (1998). The INK4a/ARF tumor suppressor: one gene--two products--two pathways. Trends Biochem. Sci..

[83] Bennett D.C. (2003). Human melanocyte senescence and melanoma susceptibility genes. Oncogene.

[84] Gray-Schopfer V.C., Cheong S.C., Chong H., Chow J., Moss T., Abdel-Malek Z.A., Marais R., Wynford-Thomas D., Bennett D.C. (2006). Cellular senescence in naevi and immortalisation in melanoma: a role for p16?. Br. J. Cancer.

[85] Rizos H., Woodruff S., Kefford R.F. (2005). p14ARF interacts with the SUMO-conjugating enzyme Ubc9 and promotes the sumoylation of its binding partners. Cell Cycle.

[86] Rizos H., McKenzie H.A., Ayub A.L., Woodruff S., Becker T.M., Scurr L.L., Stahl J., Kefford R.F. (2006). Physical and functional interaction of the p14ARF tumor suppressor with ribosomes. J. Biol. Chem..

[87] Harland M., Taylor C.F., Chambers P.A., Kukalizch K., Randerson-Moor J.A., Gruis N.A., de Snoo F.A., ter Huurne J.A.C., Goldstein A.M., Tucker M.A., Bishop D.T., Bishop J.A. (2005). Mutation hotspot at the p14ARF splice site. Oncogene.

[88] Randerson-Moor J.A., Harland M., Williams S., Cuthbert-Heavens D., Sheridan E., Aveyard J., Sibley K., Whitaker L., Knowles M., Bishop J.N., Bishop D.T. (2001). A germline deletion of p14(ARF) but not CDKN2A in a melanoma-neural system tumour syndrome family. Hum. Mol. Genet..

[89] Zuo L., Weger J., Yang Q., Goldstein A.M., Tucker M.A., Walker G.J., Hayward N., Dracopoli N.C. (1996). Germline mutations in the p16INK4a binding domain of CDK4 in familial melanoma. Nat. Genet..

[90] Goldstein A.M., Chidambaram A., Halpern A., Holly E.A., Guerry I.D., Sagebiel R., Elder D.E., Tucker M.A. (2002). Rarity of CDK4 germline mutations in familial melanoma. Melanoma Res..

[91] Mistry S.H., Taylor C., Randerson-Moor J.A., Harland M., Turner F., Barrett J.H., Whitaker L., Jenkins R.B., Knowles M.A., Bishop J.A., Bishop D.T. (2005). Prevalence of 9p21 deletions in UK melanoma families. Genes Chromosomes Cancer.

[92] Van Doorn R., Zoutman W.H., Gruis N.A. (2009). Absence of germline epimutation of the CDKN2A gene in familial melanoma. J. Invest. Dermatol..

[93] Gillanders E., Juo S.H., Holland E.A., Jones M., Nancarrow D., Freas-Lutz D., Sood R., Park N., Faruque M., Markey C., Kefford R.F., Palmer J., Bergman W., Bishop D.T., Tucker M.A., Bressac-de P.B., Hansson J., Stark M., Gruis N.A., Bishop J.N., Goldstein A.M., Bailey-Wilson J.E., Mann G.J., Hayward N., Trent J. (2003). Localization of a novel melanoma susceptibility locus to 1p22. Am. J. Hum. Genet..

[94] Kennedy C., ter Huurne. J.A., Berkhout M., Gruis N.A., Bastiaens M., Bergman W., Willemze R., Bouwes Bavinck J.N. (2001). Melanocortin 1 receptor (MC1R) gene variants are associated with an increased risk for cutaneous melanoma which is largely independent of skin type and hair color. J. Invest. Dermatol..

[95] Valverde P., Healy E., Sikkink S., Haldane F., Thody A.J., Carothers A., Jackson I.J., Rees J.L. (1996). The Asp84Glu variant of the melanocortin 1 receptor (MC1R) is associated with melanoma. Hum. Mol. Genet..

[96] Abdel-Malek Z.A., Scott M.C., Suzuki I., Tada A., Im S., Lamoreux L., Ito S., Barsh G., Hearing V.J. (2000). The melanocortin-1 receptor is a key regulator of human cutaneous pigmentation. Pigment Cell Res..

[97] Suzuki I., Tada A., Ollmann M.M., Barsh G.S., Im S., Lamoreux M.L., Hearing V.J., Nordlund J.J., Abdel-Malek Z.A. (1997). Agouti signaling protein inhibits melanogenesis and the response of human melanocytes to alpha-melanotropin. J. Invest. Dermatol..

[98] Orlow S.J., Brilliant M.H. (1999). The pink-eyed dilution locus controls the biogenesis of melanosomes and levels of melanosomal proteins in the eye. Exp. Eye Res..

[99] Chen K., Manga P., Orlow S.J. (2002). Pink-eyed dilution protein controls the processing of tyrosinase. Mol. Biol. Cell.

[100] Box N.F., Wyeth J.R., O'Gorman L.E., Martin N.G., Sturm R.A. (1997). Characterization of melanocyte stimulating hormone receptor variant alleles in twins with red hair. Hum. Mol. Genet..

[101] Rees J.L. (2003). Genetics of hair and skin color. Annu. Rev. Genet..

[102] Bastiaens M., ter Huurne J., Gruis N.A., Bergman W., Westendorp R., Vermeer B.J., Bouwes Bavinck J.N. (2001). The melanocortin-1-receptor gene is the major freckle gene. Hum. Mol. Genet..

[103] Bishop D.T., Demenais F., Iles M.M., Harland M., Taylor J.C., Corda E., Randerson-Moor J., Aitken J.F., Avril M.F., Azizi E., Bakker B., Bianchi-Scarra G., Bressac-de P.B., Calista D., Cannon-Albright L.A., Chin A.W., Debniak T., Galore-Haskel G., Ghiorzo P., Gut I., Hansson J., Hocevar M., Hoiom V., Hopper J.L., Ingvar C., Kanetsky P.A., Kefford R.F., Landi M.T., Lang J., Lubinski J., Mackie R.M., Malvehy J., Mann G.J., Martin N.G., Montgomery G.W., van Nieuwpoort F.A., Novakovic S., Olsson H., Puig S., Weiss M., van W.W., Zelenika D., Brown K.M., Goldstein A.M., Gillanders E.M., Boland A., Galan P., Elder D.E., Gruis N.A., Hayward N.K., Lathrop G.M., Barrett J.H., Bishop J.A. (2009). Genome-wide association study identifies three loci associated with melanoma risk. Nat. Genet..

[104] Brown K.M., Macgregor S., Montgomery G.W., Craig D.W., Zhao Z.Z., Iyadurai K., Henders A.K., Homer N., Campbell M.J., Stark M., Thomas S., Schmid H., Holland E.A., Gillanders E.M., Duffy D.L., Maskiell J.A., Jetann J., Ferguson M., Stephan D.A., Cust A.E., Whiteman D., Green A., Olsson H., Puig S., Ghiorzo P., Hansson J., Demenais F., Goldstein A.M., Gruis N.A., Elder D.E., Bishop J.N., Kefford R.F., Giles G.G., Armstrong B.K., Aitken J.F., Hopper J. L., Martin N.G., Trent J.M., Mann G.J., Hayward N.K. (2008). Common sequence variants on 20q11.22 confer melanoma susceptibility. Nat. Genet..

[105] Gudbjartsson D.F., Sulem P., Stacey S.N., Goldstein A.M., Rafnar T., Sigurgeirsson B., Benediktsdottir K.R., Thorisdottir K., Ragnarsson R., Sveinsdottir S.G., Magnusson V., Lindblom A., Kostulas K., Botella-Estrada R., Soriano V., Juberias P., Grasa M., Saez B., Andres R., Scherer D., Rudnai P., Gurzau E., Koppova K., Kiemeney L.A., Jakobsdottir M., Steinberg S., Helgason A., Gretarsdottir S., Tucker M.A., Mayordomo J.I., Nagore E., Kumar R., Hansson J., Olafsson J.H., Gulcher J., Kong A., Thorsteinsdottir U., Stefansson K. (2008). ASIP and TYR pigmentation variants associate with cutaneous melanoma and basal cell carcinoma. Nat. Genet..

[106] Falchi M., Bataille V., Hayward N.K., Duffy D.L., Bishop J.A., Pastinen T., Cervino A., Zhao Z.Z., Deloukas P., Soranzo N., Elder D.E., Barrett J.H., Martin N.G., Bishop D.T., Montgomery G.W., Spector T.D. (2009). Genome-wide association study identifies variants at 9p21 and 22q13 associated with development of cutaneous nevi. Nat. Genet..

